# A reply to Nieberding and Holveck: beyond experimental design and proximate mechanisms - mate choice in the face of sexual conflict

**DOI:** 10.1186/s12983-017-0242-9

**Published:** 2018-04-27

**Authors:** Klaus Fischer, Isabell Karl, Ian A. N. Dublon, Tobias Kehl

**Affiliations:** 1 Zoological Institute and Museum, Greifswald University, Loitzer Straße 26, D-17489 Greifswald, Germany; 2 Infrastrukturavdelningen, Swedish University of Agricultural Sciences, Slottsvägen 1, P.O. Box 19, SE-230 53 Alnarp, Sweden

**Keywords:** *Bicyclus anynana*, Courtship behaviour, Female mate choice, Honest signal, Lepidoptera, Male sex pheromone, Mating success, Residual reproductive value, Sexual conflict, Experimental constraints

## Abstract

We summarise our work on male mating behaviour in the tropical butterfly *Bicyclus anynana*, responding to the commentary provided by Nieberding and Holveck. We acknowledge that our laboratory studies are not free of shortcomings and potential caveats, though we attempted to address or highlight these within each paper. The concerns raised seem to stem mainly from different notions with respect to the proximate basis of old male mating advantage, and specifically the relative importance of male behaviour versus pheromone blend. In our view, our experiments provided compelling evidence for a prominent role of male behaviour, while we were unable to obtain clear evidence for a major role of male sexual pheromones. In addition to the lack of evidence we argue that a preference of females for older males based on pheromone blend is unlikely, as pheromone titres do not seem to indicate male quality and, more importantly, females actually suffer a fitness cost when mating with older males. The latter suggests that old male mating advantage arises from sexual conflict rather than cooperation. We thus highlight the importance of considering both the proximate and the ultimate level for gaining an integrative understanding of complex behavioural patterns.

## Background

In their contribution, Nieberding and Holveck [1], focussing on Kehl et al. 2015 [2] express their concerns about our work on factors affecting male mating success in the tropical butterfly *Bicyclus anynana* (Butler, 1879) [2–10]. While using our work to develop their ideas, their considerations may be of general importance to many laboratory-based behavioural studies. We therefore appreciate their comments highlighting matters to be considered in a variety of experimental contexts. In essence, Nieberding and Holveck address an old but still highly valid issue pertinent to ecological and ethological research: the tension between the demand for high levels of control and replication, as can be typically achieved in laboratory settings only, and ecological realism. The latter can basically be assured in field studies only, where conditions are evidently difficult or indeed impossible to control and manipulate. Obviously, there are pros and cons to each approach, and evaluating these and addressing limitations of laboratory ethology are beyond the scope of this contribution. In short, as correctly noted by Nieberding and Holveck, our experiments aimed to optimise levels of control and replication, which resulted in experimental conditions quite far removed from natural situations. Thus, we whole-heartedly agree that one needs to be cautious when interpreting the results of such laboratory studies, especially when trying to extrapolate back towards natural situations. Before commenting on the three concerns raised by Nieberding and Holveck, we will provide some hopefully helpful background information.

## Old male mating advantage in the butterfly *Bicyclus anynana*

In 2008, our group published a study on old male mating advantage in *B. anynana* [3]. Subsequently, we were intrigued by the factors affecting male mating success in this particular organism, which has prompted the series of publications mentioned by Nieberding and Holveck ([2–10]; see also [11–13]). We predicted the occurrence of old male mating advantage based on the lower residual reproductive value of older males, favouring more aggressive and risky mating behaviour (representing a form of terminal investment; [3]). Thus, we explicitly developed our hypotheses within a life-history theory framework, a perspective rarely adopted in behavioural ecology and studies on sexually selected characters [14, 15]. Based on the sound theoretical framework used, it is difficult for us to see why our results should be ‘surprising’ rather than expected (see further below). Since the first study mentioned above, the pattern of old male mating advantage has been repeatedly demonstrated in *B. anynana* [3, 5, 8, 10, 13], and, as far as we can see, the pattern as such is not debated. Different notions, however, exist with respect to the proximate basis of this intriguing pattern. While we, after having performed a series of experiments trying to disentangle different hypotheses, favour a prominent role of male behaviour [3, 5, 8, 10], Nieberding and Holveck favour female mate choice based on male pheromone blend [1, 12]. Obviously, both explanations are not mutually exclusive. Nevertheless, much of the concerns raised seem to stem from different notions regarding the *relative importance* of male behaviour versus pheromone blend on male mating success, and specifically old male mating advantage, in *B. anynana*.

## First concern: Use conditions that allow females to escape

Indeed, we have used, for practical reasons, unnaturally high densities in our experiments [2–10, 13], which is probably at least to some extent always the case in laboratory-based behavioural studies. Please note though that, unlike stated by Nieberding and Holveck, (1) information on cage size is given in [2] (cf. page 7), (2) the highest densities mentioned were used in *one* experiment only and in order to induce differential effects of density prior to, rather than during mating trials [4], and that (3) mating trials were conducted over eight consecutive days in one experiment only to investigate female mating frequency (thus a long period was evidently needed) rather than male mating success [5]. We agree that experimental conditions may affect patterns of mate choice and mating success, which may lead to an over- or underestimation of the impact of male behaviour. However, as acknowledged by Nieberding and Holveck, we explicitly stated this caveat [5], and note that in other lepidopteran behavioural studies small cage sizes have been used as well [16–18].

With respect to cage size, it might be interesting to note that we actually investigated the effects of small cages versus more natural settings on mating outcomes in *B. anynana* [3]. We showed that old male mating advantage tended to diminish in larger flight cages as opposed to small cages [3]. This reinforces rather than discounts a vital importance of male courtship behaviour on male mating success. Indeed, if pheromone blend was the crucial cue guiding females to the preferred male at this scale, old males should retain a mating advantage irrespective of cage size, which was not the case. Thus, the pattern found suggests an important role of male behaviour. We suppose that old male mating advantage may diminish in more natural settings, because under such conditions age-diminished (flight) ability becomes more important [3].

We maintain though that female mate choice cannot be dismissed in butterflies, irrespective of what the experimental conditions are. As noted by ourselves and Nieberding and Holveck [1], males seem to be unable to enforce copulation, thus females do not need to escape from males but possess different behaviours to prevent mating with non-preferred males [19]. Consequently, while male butterflies initiate courtship, it is the female that ultimately decides with whom she will mate. These considerations hold at least for wet season butterflies, which have been used here, while matters may be different in the dry season [20, 21]. Therefore, there is absolutely no dissent with respect to the crucial role of female choice, which will always remain the most crucial factor determining butterfly male mating success, but rather only with respect to the cues that females may base their decision upon (e.g. male pheromone blend or aggressiveness). Please note the apparent misunderstanding in Nieberding and Holveck [1] with respect to male courtship activity. We did not refer to activity in male-male contests as stated, but to courtship activity (e.g. [2]).

## Second concern: produce treatments that allow females to be choosy

We also agree with the notion that relevant treatments should be used that allow females to be choosy. However, we were a bit puzzled when reading the first paragraph of the according section in [1], insisting that older butterflies expressing larger variation in male sex pheromones (MSPs) should have been used in [2]. We explicitly and repeatedly mentioned (including in the title) that our study focusses solely on young (though sexually active) males [2]. Thus, any conclusions that can be drawn from this study are naturally restricted to these. This study did not attempt to investigate any age-specific variation, but only tested for a covariation between pheromone titre and (1) mating success and (2) other fitness-related traits, which we did not find. We even suggested that future studies should test whether these results also hold true in older males [2]. Please further note that we exclusively focussed on natural (and therefore ecologically relevant) sex pheromone variation here. In contrast to Nieberding and Holveck’s notion, our data clearly show that 2-day old males are not homogeneous with respect to male sex pheromone composition, but instead differ not insubstantially ([2]; see also [8]). Finally, we maintain that using two day-old sexually capable males is ecologically relevant. While *B. anynana* may indeed live up to several months especially under laboratory conditions, this is basically true for the dry season during which the butterflies do not reproduce [22, 23]. Therefore, the relevant season for investigating mating behaviour is the wet season during which butterflies are reproductively active. Here, mean longevity can be expected to be much shorter, given that random mortality is common in insects [24, 25].

With respect to our other study [8], which was also mentioned, in which we indeed investigated age-specific variation, we unfortunately fail to see the support for several claims articulated by Nieberding and Holveck. First, we see no evidence for the claim that “young and old perfumes did not differ anymore in composition, and contained only trace amounts of hexadecanal, at the time they were applied” [1]. We did not carry out such measurements, and our work in [8] does suggest that perfumes showed clear differences. Regrettably, we could also not find the data presented in Nieberding and Holveck in our text, so we cannot comment on the values provided. What we did measure in the respective publication, experiment 2, is the on-wing pheromone composition after death, i.e. after the 1.5 h mating experiments [8]. At this stage, only MSP3 and the total amount of MSP differed among treatment groups, but not MSP1 and MSP2 [8]. Again, this caveat was explicitly stated in our paper: “Therefore the present results need to be interpreted with caution… The absence of variation in MSP2 titers at the end of our experiments may have prevented females from using this particular cue assumed to be of special importance” [8]. We concluded from all the data obtained in this study that pheromone signals indeed seem to be involved in male mating success, but several lines of evidence suggested a stronger impact of male behaviour [8]. In our experiments, for instance, old male mating advantage persisted despite similar pheromone blends, which, as stated above, suggests that other factors are likely to be more important. While MSPs have been shown via removal / reapplication studies to influence male mating success [8, 24], in the context of old male mating advantage, there is to our best knowledge thus far no experimental evidence for a prominent role of MSP2. This is because all three MSPs were manipulated simultaneously in previous re-application experiments [26]. The argument of a particular importance of MSP2 exclusively rests on its large relative variation with age, but note that both other MSPs are far more abundant [26]. We suggest that the principal role of MSPs in *Bicyclus* is species-recognition as supported by several studies [27, 28].

## Third concern: multimodality of mate choice

We whole-heartedly agree with this point, namely that female mate choice is typically based on several traits and thus multimodal, and that the use of one cue does not exclude the use of others and have never suggested otherwise. Investigating this complexity was exactly the aim of our work, by targeting several traits at a time and evaluating their relative roles. Over the last years our group has investigated a substantial number of traits which may be related to mate choice in *B. anynana*, including pheromone blend, male and female behaviour, relatedness, social factors (e.g. sex ratio, density), morphology (body size, eyespot size), physiology (fat content), and reproductive traits (sperm numbers, spermatophore size) [2–13]. We even evaluated potential fitness consequences for the mated females [10]. Based on this array of studies we hope to convince the readership that we fully appreciate the multimodality of mate choice. Male behaviour is surely one such trait affecting female decisions. We further suggested that, at least in specific situations, it may be even the most important one [3, 6, 8, 10], which does of course not exclude that other traits (e.g. UV reflectance of eyespots [29]) are additionally used and / or may be of overriding importance in other contexts.

## Testing hypothesis and evaluating the evidence

In relation to the concerns raised by Nieberding and Holveck, we would like to stress a fundamental point. In the end, science is about testing hypotheses by evaluating (experimental) evidence. In the context of male mating success we formulated an array of alternative hypotheses over the last years, which we tested in controlled laboratory settings. Of course, such laboratory experiments can always and indeed should be objectively criticised, stressing the need (1) to interpret results in a cautious manner and (2) to carefully describe the methods used, and (3) that in the first place all experimental results are valid under the conditions under which they were obtained. We are after all working with a highly tractable insect model [30], where for example rearing temperature, photoperiod, relative humidity, and diet are all standardised in ways not possible in nature. For example, we tested alternative hypotheses regarding the role of male pheromone blend versus male behaviour. To this end, we manipulated male pheromone blend and / or female scent organs [6, 8, 10]. What can be predicted? We hypothesised that, if female mate choice based on pheromone blend would be the principal cue, old male mating advantage should not persist when males of different age smell similar or when females are unable to smell. If male behaviour, however, was most important in shaping old male mating advantage, the pattern should persist regardless of pheromone or female manipulation. The results showed that old male-mating advantage persisted throughout.

## Beyond experimental design and proximate mechanisms

Beyond the mechanisms involved in male mating success, though, the more intriguing question to our mind is why females should ultimately prefer males expressing some specific secondary sexual trait, e.g. a specific pheromone composition? Or why should they preferentially mate with older males? Amongst others, older males are in poor condition and more likely to have mated already, thus providing less nutrients and sperm of inferior quality [3, 10, 12]. The only argument we can think of is a sort of good genes hypothesis based on the proven survival abilities of older males [6, 8, 10]. If so, male sex pheromones may function as honest signals indicating male age and thereby quality. How likely though is this scenario? The principal question here is whether pheromones are costly to produce. If not, inferior males are not expected to ‘voluntarily’ signal their poor quality, but rather to cheat. Clearly, much more work needs to be done on answering these issues, but at least it seems conceivable that vigorous and aggressive courtship may provide a more reliable cue to females than pheromone blend. At present, there is, to the best of our knowledge, no evidence that *B. anynana* females may benefit from mating with males of a specific pheromone blend. In our study using young males, pheromone titres were not associated with any fitness-related female or offspring trait measured [2] though this does not preclude the possibility that there are no such traits or that matters differ for older males, which remains to be tested. More intriguingly, we provided experimental evidence that females actually suffer from mating with older males [10, 12]. How does this result fit to an active female preference for older males?

For the time being, we suggest the following with respect to old-male mating advantage in the *B. anynana* system. (1) Male aggressiveness during contests and courtship is a positively selected trait in *B. anynana*, as is the case in other butterflies [14, 31]. (2) Females prefer the most vigorous, persistent and aggressive males (in order to produce vigorous sons, similar to the sexy son hypothesis), or perhaps they simply decide to commence copulation to avoid the time and energy costs involved in rejecting aggressive males. (3) We assume that, in general, there is a positive correlation between male condition and aggressiveness; thus, females prefer males of good condition. (4) Apart from males in good shape, though, older males also show vigorous courtship, as a result of their low residual reproductive value. (5) Old male mating advantage results as a consequence of point (2), but actually reflects sexual conflict rather than cooperation as such males are ‘preferred’ either mistakenly or as a matter of convenience, which is supported by detrimental effects of mating with older males on egg hatching success [10, 12]. Note that the fact that courtship activity increases with increasing age is, as far as we know, undebated [3, 4, 6, 8, 10].

## Conclusions: Beyond sex as a harmonious event

In summary, we welcome the critique of Nieberding and Holveck [1] and we agree that being aware of the experimental environment is very important in all behavioural studies. We accept that our studies, like others are not free of shortcomings and potential caveats, however every attempt has been made to explicitly address these within our papers. Nevertheless, we provided, to our mind, persuasive evidence for a prominent role of male behaviour on mating success, while we found no clear evidence for a major role of male sexual pheromones beyond species recognition. However, we would like to encourage further constructive discourse and multidisciplinary studies from various fields of expertise on these issues. We argue that a fully integrative understanding of mating behaviour requires considering (1) alternative hypotheses, (2) the proximate as well as the ultimate level, and (3) benefits from adopting a life-history perspective. We believe that the different notions regarding the relative importance of male behaviour stem at least partly from differential views on conflict and cooperation between the sexes. To our mind, we should abolish the idea of mating being seen as a largely harmonious event governed by common interests [32, 33]. Rather, the overriding importance of sexual conflict and individual interests should be acknowledged, which will perhaps affect the interpretation of empirical evidence. To summarise, in the *Bicyclus anynana* system currently (1) no benefits of mating with males of a specific pheromone blend or older rather than younger males are known, (2) in contrast females suffer fitness costs when mating with older males, and that (3) there can be no doubt that older males are indeed more aggressive than younger males. At this point, we would like to leave it to the readership to decide whether our results are indeed surprising, as stated by Nieberding and Holveck [1], or actually well in line with what could be envisaged.
